# Reliability and Validity of the Workplace Social Distance Scale

**DOI:** 10.5539/gjhs.v7n3p46

**Published:** 2014-10-28

**Authors:** Hatsumi Yoshii, Nozomu Mandai, Hidemitsu Saito, Kouhei Akazawa

**Affiliations:** 1 Health Sciences, Tohoku University, Graduate school of Medicine, Miyagi, Japan; 2 Department of Medical Informatics and Statistics, Niigata University Graduate School of Medicine, Japan

**Keywords:** social distance, workplace, scale, reliability, validity

## Abstract

Self-stigma, defined by a negative attitude toward oneself combined with the consciousness of being a target of prejudice, is a critical problem for psychiatric patients. Self-stigma studies among psychiatric patients have indicated that high stigma is predictive of detrimental effects such as the delay of treatment and decreases in social participation in patients, and levels of self-stigma should be statistically evaluated. In this study, we developed the Workplace Social Distance Scale (WSDS), rephrasing the eight items of the Japanese version of the Social Distance Scale (SDSJ) to apply to the work setting in Japan. We examined the reliability and validity of the WSDS among 83 psychiatric patients. Factor analysis extracted three factors from the scale items: “work relations,” “shallow relationships,” and “employment.” These factors are similar to the assessment factors of the SDSJ. Cronbach’s alpha coefficient for the WSDS was 0.753. The split-half reliability for the WSDS was 0.801, indicating significant correlations. In addition, the WSDS was significantly correlated with the SDSJ. These findings suggest that the WSDS represents an approximation of self-stigma in the workplace among psychiatric patients. Our study assessed the reliability and validity of the WSDS for measuring self-stigma in Japan. Future studies should investigate the reliability and validity of the scale in other countries.

## 1. Introduction

Studies on external attitudes toward psychiatric patients have been conducted across the world (Corrigan et al., 2012; Haraguchi et al., 2009; Lysaker et al., 2007; Whatley, 1959). These studies of external attitudes have investigated social distance and stigma by measuring the distance and level of emotion a subject expresses toward psychiatric patients. Higher levels of social distance and stigma have negative effects on psychiatric patients (Lysaker et al., 2007; Heather et al., 2001; Kadri et al., 2005) and can also affect their quality of life and treatment. For example, having negative perceptions of mental illness is one reason for delayed treatment (Lysaker et al., 2007; Esterberg et al., 2008; Tanaka et al., 2003). Psychiatric patients might not be open about their disease or avoid seeking treatment, and these behaviors can also have harmful effects on their lives (Corrigan et al., 2012) and lead to discrimination in education, employment, personal relationships, marriage, and housing (Takahashi et al., 2009). Around the world, social distance and stigma research has found a relationship between attitude and the course of illnesses such as bipolar disorder (Hawke et al., 2013), bulimia nervosa (McLean et al., 2013), ADHD, and depression (Ohan et al., 2013).

Self-stigma is defined as having a negative attitude toward oneself and being conscious of being a target of prejudice (Corrigan et al., 2009; Watson et al., 2007). After psychiatric patients become ill, they are likely to view themselves as discriminated against and, in fact, to suffer discrimination, in addition to experiencing the pain of the disease. For this reason, several studies on self-stigma have been conducted. Girma et al. (2013) found that, in Ethiopia, women exhibited higher self-stigma than men. They also found that both drug side effects and perceived signs of mental illness were associated with increased self-stigma, whereas education and self-esteem were observed to decrease self-stigma among people with mental illness. In a study in Croatia, Israel, Lithuania, Malta, Romania, and Sweden, Krajewski, Burazeri, and Brand (2013) found that 33% of people with a psychiatric disorder had moderate to high self-stigma scores. Significant predictors of high self-stigma scores were being aged 50–59, holding current employment, and having a lower level of social contact. Also, in a survey on quality of life among psychiatric patients in Sweden, Bejerholm and Björkman (2011) found that higher empowerment scores were associated with fewer symptoms and experiences of stigma, a higher level of engagement in daily activities and community life, better quality of life, and more participation in work rehabilitation. These findings led us to hypothesize that psychiatric patients in employment experience work-related self-stigma.

Work-related questions are rarely found in assessments of stigma for people with psychiatric disorders. The Link Devaluation-Discrimination Measure (Link, 1987) consists of 12 items graded on a four-point Likert scale, with higher scores representing increased self-stigma. This measure includes three items related to work: “Most people would not hire a former mental patient to take care of their children, even if he or she had been well for some time;” “Most employers will hire a former mental patient if he or she is qualified for the job;” and “Most employers will pass over the application of a former mental patient in favor of another applicant.” The Internalized Stigma of Mental Illness (ISMI) scale includes a total of 29 items graded on a four-point Likert scale ranging from 1 (*strongly agree*) to 4 (*strongly disagree*) and comprises five subscales (Ritsher et al., 2003). This scale has no work-related questions. Higher scores on the ISMI indicate higher self-stigma. The reliability values (Cronbach’s alpha) for the five subscales and the overall scale were determined as follows: alienation (6 items)=0.84, stereotype endorsement (7 items)=0.73, discrimination experience (5 items)=0.79, social withdrawal (6 items)=0.77, stigma resistance (5 items)=0.65, and overall self-stigma=0.89 (Girma et al., 2013).

Because work-related items are rarely found on assessment scales, it is unclear whether psychiatric patients typically experience work-related self-stigma. To address the need for a work-related assessment scale, in the present study, we rephrased the eight items of the Social Distance Scale, Japanese version (SDSJ) to apply to the work setting and then administered the new scale to a sample of psychiatric patients.

## 2. Methods

### 2.1 Subjects

Questionnaires were distributed to 116 stable psychiatric patients who were either outpatients or inpatients in an open ward of a psychiatric hospital in Japan. Respondents were invited to complete the questionnaire by the doctor. The participants were limited to those who had worked after beginning psychiatric treatment.

### 2.2 Ethical Considerations

The participants were told that the content of the investigation would be used for research only, that they could withdraw from the study at any time, that their participation would be kept confidential, and that all data would be handled anonymously. Approval to conduct this research was obtained from the psychiatric hospital and the Graduate School of Medicine, Tohoku University, Japan.

### 2.3 Measures

Two questionnaires were administered to the psychiatric patients in Japan: the SDSJ and the newly developed Workplace Social Distance Scale (WSDS). The SDSJ, an 8-item inventory (Makita, 2006) adapted from the Whatley Social Distance Scale (Whatley, 1959), was designed to measure social distance toward schizophrenia. It is a self-report inventory that can be used by psychiatric nurses and medical students, as it was modified by rephrasing portions of the original scale. It contains a relatively small number of questions, including two work-related questions. Makita (2006) created the Japanese version and verified its validity in Japan, obtaining a Cronbach’s alpha value of 0.849, which indicates good reliability. This scale is used solely in Japan (Haraguchi et al., 2009; Yoshii et al., 2012). The SDSJ’s two work-related items are: “I would rather not hire a person with schizophrenia who had been in a hospital” and “If I needed a baby sitter, I would be willing to hire a woman with schizophrenia.” For each of the eight items, respondents were asked to report how often each statement is true on a scale ranging from 1 (0 points) to 4 (3 points). Totaled responses resulted in a score ranging from 0 to 24, with higher scores indicating higher levels of social distance.

The WSDS was created by modifying the SDSJ, changing the eight SDSJ items to apply to employed psychiatric patients. The scale was designed to measure self-stigma that psychiatric patients feel when they work. The WSDS, like the SDSJ, is an 8-item self-report inventory with a total score ranging from 0 to 24 points (Table 1).

**Table 1:** Factor Analysis of the Workplace Social Distance Scale Item Scores

Item	Item Content	Factor Loadings			Communality

		Work Relations	Shallow Relationships	Employment	
Factor I (Work relations)					
3	It would bother me to work next to a coworker with psychosis.	0.92	0.09	−0.27	0.804
1	It is best not to associate with a coworker with psychosis who has been in a mental hospital.	0.54	−0.21	0.32	0.433
6	Bosses with psychosis should not be allowed to teach how to work at the workplace.	0.45	0.19	0.12	0.374

Factor II (Shallow relationships)					
8	I would be against any secretary of mine marrying a man with psychosis.	0.09	0.8	0.07	0.75
4	I would not ride in a car driven by a coworker with psychosis.	−0.09	0.53	0.29	0.36

Factor III (Employment)					
5	I would rather not hire a person with psychosis who had been in a hospital.	0.17	0.08	0.76	0.744

Omitted Questions					
2	It is wrong to shy away from a coworker with psychosis.	−0.09	0.16	0.23	0.066
7	If I needed a babysitter at the in-house nursery, I would be willing to hire a woman with psychosis.	−0.06	0.06	0.20	0.04

### 2.4 Analytical Methods

Statistical analysis was performed using SPSS, version 18. Descriptive data analysis was conducted by calculating frequencies, mean scores, and standard deviations. The method of factor analysis was principal axis factoring with promax rotation. The internal consistency of the WSDS was tested using Cronbach’s alpha coefficient, and the reliability of the instrument was tested using the split-half technique. Pearson’s correlation coefficient was used to examine the relationship between the WSDS and the SDSJ.

## 3. Results

### 3.1 Participant Characteristics

In total, we obtained valid responses from 83 patients. The participants were 42 patients with schizophrenia (50.6%), 15 with depression (18.1%), 7 with alcohol-related disorders (8.4%), 2 with bipolar disorder (2.4%), 14 with other disorders (16.9%), and 3 with uncertainty (3.6%). The sample consisted of 62 men (74.7%) and 21 women (25.3%). Sixty-four participants (77.1%) had been hospitalized in a psychiatric hospital, and 19 (22.9%) had not been hospitalized in a psychiatric hospital. Thirty-six participants (43.4%) reported being treated differently at work because of their disease, 42 participants (50.6%) related no such experiences, and the experiences of 5 (6.0%) were unknown.

### 3.2 WSDS

Factor analysis of the WSDS was based on data obtained from the responses of the 83 psychiatric patients. After extracting the factors, items 2 and 7 were found to have communalities near 0, so these questions were excluded, leaving six remaining questions in the WSDS (Table 1).

The mean score on the WSDS for psychiatric patients was 6.27 points (*SD* = 0.67), and the data displayed a normal distribution. No significant differences in WSDS test scores were observed by gender or inpatient experience.

A scree plot of eigenvalues indicated that a three-factor model was reasonable. Using principal axis factoring with promax rotation, on the basis of the component items, three factors emerged and were labeled “work relations,” “shallow relationships,” and “employment” (Table 1). The reliability of the WSDS was calculated as an index of internal consistency (Cronbach’s alpha). Cronbach’s alpha was 0.753 overall, 0.662 for the work relations subscale, and 0.649 for the shallow relationships subscale. There was no Cronbach’s alpha for the employment subscale (Table 2). The split-half method yielded a reliability coefficient of 0.801 for the returned questionnaires.

**Table 2:** Internal Consistency of the Workplace Social Distance Scale

		Cronbach’s alpha
Total scores		0.753
Subscales		
	Factor I (Work relations)	0.662
	Factor II (Shallow relationships)	0.649
	Factor III (Employment)	-

### 3.3 WSDS and SDSJ

Cronbach’s alpha for the SDSJ was 0.781 overall for the 83 respondents, and we found a significant positive correlation between the total scores on the SDSJ and WSDS (*r*=0.626, *p*<0.001; Table 3). In terms of the relationship between the SDSJ and WSDS subscales, we observed positive correlations between the SDSJ and work relations (*r*=0.464, *p*<0.001), shallow relationships (*r*=0.565, *p*<0.001), and employment (*r*=0.483, *p*<0.001).

**Table 3:** Correlations between the WSDS and the SDSJ

	Total Scores	WSDS Factor I (Work Relations)	WSDS Factor II (Shallow relationships)	WSDS Factor III (Employment)
**SDSJ**	0.626*	0.464*	0.565*	0.483*

*Note.* WSDS = Workplace Social Distance Scale; SDSJ = Social Distance Scale, Japanese version.

* *p* < 0.001.

## 4. Discussion

### 4.1 Reliability of the WSDS

In this study, we rephrased the items of the SDSJ to be relevant to the work setting. We then surveyed a sample of psychiatric patients to study the reliability and validity of the newly developed scale measuring work-related self-stigma. The overall Cronbach’s alpha coefficient was 0.781 for the SDSJ and 0.753 for the WSDS. Factor analysis indicated that the appropriate number of items was six, with a three-factor structure. The three factors extracted from the WSDS were defined as individual subscales: work relations, shallow relationships, and employment. Cronbach’s alpha was 0.662 for work relations, but it was somewhat lower (0.649) for shallow relationships. Reliability was obtained for total scores, and the lack of reliability must be examined more closely. To confirm the reliability of the WSDS, we need to examine larger samples and samples from other geographical locations. The value for split-half reliability was also high (0.801). Taken together, these results generally prove the reliability of the WSDS.

### 4.2 Validity of the SDSJ and WSDS

Two of the WSDS factors extracted in this study, work relations and shallow relationships, were similar to the factors of the SDSJ (Yoshii et al., 2012). The SDSJ assesses social distance through eight items asking about private and social relationships related to schizophrenia. The WSDS subscales extracted by factor analysis approximated items on the SDSJ for the assessment of social distance.

In a survey of parents with children in junior high or high school, gender and participation in activities for the welfare of people with mental illnesses were significantly associated with these two factors on the SDSJ (Yoshii et al., 2012). However, no significant social or demographic factors were extracted for the WSDS. We need to examine more samples to clarify the reason for this difference.

We found a positive correlation between total WSDS and SDSJ scores. In addition, we observed a positive correlation between the SDSJ and each of the three WSDS subscales (work relations, shallow relationships, and employment). These results suggest the concurrent validity of the WSDS.

### 4.3 Challenges

Haraguchi et al. (2009) reported a difference in social distance toward patients with schizophrenia among medical staff in Japan and China. Clearly, cultural differences gave rise to such dissimilarities on social distance. Self-stigma might be also similar. Although we have demonstrated that the reliability and validity of the WSDS have been proven among Japanese psychiatric patients, these issues have not been investigated in other countries. It is crucial for us to determine whether the WSDS is also reliable and valid outside Japan. In addition, the factor of work-related self-stigma has not been identified, and further study with many participants using the WSDS is required. In the present study, as part of the WSDS, psychiatric patients were asked about their views on the item “It is best not to associate with a coworker with psychosis who has been in a mental hospital.” Thus, the content of the WSDS items represents how employed psychiatric patients feel. Therefore, our study might represent tendencies in the emotional attitudes of individual psychiatric patients more strongly because of their awareness of their disease. Consequently, we can effectively use the WSDS when patients have trouble starting or continuing employment. After sharing the evidence that “self-stigma disturbs employment” with patients, the WSDS can be used in two ways. First, the patient can be informed of his/her stigma score, allowing him/her to compare their own score with the average figure or with other people’s scores. Second, by reviewing the individual scores on the WSDS questions, it is possible to select the items with high self-stigma scores and to then conduct focused cognitive behavioral therapy.

In the future, the WSDS should be applied to investigate the stigma of employers toward psychiatric patients. A promising next step would then be to investigate the relationship between the self-stigma of psychiatric patients and the stigma of their employers.

## 5. Conclusion

Our study assessed the reliability and validity of the WSDS for measuring self-stigma in Japan. Future studies should investigate the reliability and validity of the scale in other countries.
